# Dynamic Mechanical Alignment in Total Knee Arthroplasty for Varus Morphotypes: A Retrospective Study of a Manual Technique and Midterm Outcomes

**DOI:** 10.7759/cureus.117845

**Published:** 2026-10-13

**Authors:** Jitesh K Jain, Ruchi Goyal, Harish Bansal

**Affiliations:** 1 Orthopedics and Traumatology, RHL Rajasthan Hospital, Jaipur, IND; 2 Orthopedics and Robotic Joint Replacement, RHL Rajasthan Hospital, Jaipur, IND

**Keywords:** coronal plane alignment, dynamic mechanical alignment, functional alignment, soft-tissue balance, total knee arthroplasty, varus morphotype

## Abstract

Background: This study evaluated the midterm clinical and radiological outcomes of a manual dynamic mechanical alignment (DMA) technique for primary total knee arthroplasty (TKA) in knees with varus osteoarthritis. Materials and methods: This single-center retrospective observational study included 300 patients (542 knees) who underwent primary TKA using a manual DMA algorithm between July 2022 and July 2023. Clinical and radiological records were reviewed through July 2025, providing 24-36 months of overall follow-up. The primary analysis reported range of motion (ROM), Knee Society Score (KSS), Western Ontario and McMaster Universities Osteoarthritis Index (WOMAC), and radiological parameters, which correspond to the standardized 12-month postoperative assessment, whereas pain was assessed using the VAS at one month. Outcomes also included coronal plane alignment of the knee (CPAK) classification, arithmetic hip-knee-ankle angle, joint-line obliquity, and postoperative complications. Results: Mean ROM increased from 95.66 ± 11.56° preoperatively to 133.82 ± 7.69° at 12 months. Mean KSS increased from 24.7 ± 14.63 to 85.58 ± 8.35, while mean WOMAC decreased from 62.62 ± 7.01 to 10.82 ± 13. All 542 knees achieved the intended physiologic mediolateral balance pattern without superficial medial collateral ligament (sMCL) release, and 418 knees (77.12%) retained their preoperative CPAK class. Conclusions: In this retrospective cohort, manual DMA improved clinical outcome measures at the standardized 12-month assessment, and it achieved the intended physiologic mediolateral balance pattern without sMCL release. These findings demonstrate technical feasibility but do not establish superiority over other alignment strategies.

## Introduction

Total knee arthroplasty (TKA) is an established treatment for advanced knee osteoarthritis, and achieving appropriate limb alignment together with balanced soft tissues is important for satisfactory postoperative function. Conventional mechanical alignment (CMA) aims to position the femoral and tibial components perpendicular to their respective mechanical axes, restoring a neutral hip-knee-ankle (HKA) axis. Although CMA has demonstrated reliable implant survivorship, some patients remain dissatisfied after TKA, suggesting that restoring uniform neutral alignment may not reproduce the native anatomy and kinematics of every knee [1].

Consequently, several alternative alignment strategies have been proposed. Anatomic alignment was introduced to reproduce physiologic joint-line obliquity while maintaining overall mechanical alignment of the limb [2]. Kinematic alignment (KA) subsequently sought to restore the patient's pre-arthritic joint surfaces and native axes by matching component position and bone resection to individual anatomy [3]. Concerns regarding excessive component varus or valgus with unrestricted KA led to the development of restricted KA, in which individualized positioning is maintained within predefined alignment boundaries [4,5]. Regardless of the alignment strategy, appropriate flexion-extension balance and mediolateral soft-tissue stability remain important determinants of postoperative function and patient satisfaction [6,7].

Functional alignment (FA) has more recently emerged as an individualized approach in which implant position and bone resections are modified according to the patient's anatomy and soft-tissue envelope to obtain balanced flexion and extension gaps while minimizing ligamentous release [8-11]. Contemporary FA techniques are commonly performed with robotic-assisted systems, which permit detailed intraoperative assessment and adjustment of component position. However, dependence on robotic technology may limit widespread implementation in centers where such systems are unavailable or impractical. The biomechanical principles underlying individualized alignment and gap balancing may still apply to conventional manual TKA.

Dynamic mechanical alignment (DMA) is a manual bone-modulation approach that incorporates patient-specific soft-tissue balance while maintaining restricted mechanical alignment boundaries. In this technique, the distal femoral resection, femoral component rotation, proximal tibial coronal resection, and posterior tibial slope are adjusted based on intraoperative gap assessment to minimize extensive ligamentous releases. The technique combines individualized balancing with the reproducibility and accessibility of manual instrumentation.

This study aimed to describe the operative principles and surgical technique of manual DMA and to evaluate the postoperative clinical and radiological outcomes in patients with varus knee osteoarthritis undergoing primary TKA. The principal clinical outcomes evaluated were range of motion (ROM), Knee Society Score (KSS), Western Ontario and McMaster Universities Osteoarthritis Index (WOMAC), and pain assessed using the Visual Analog Scale (VAS). Secondary descriptive outcomes included arithmetic HKA (aHKA) angle, joint-line obliquity (JLO), coronal plane alignment of the knee (CPAK) classification, postoperative complications, and the intraoperative balancing endpoint. Because this retrospective, single-center study lacked a comparator group, it was designed to evaluate technical feasibility and postoperative associations rather than comparative effectiveness or superiority over other alignment strategies.

## Materials and methods

Study design, setting, and duration

This single-center retrospective observational study reviewed 300 patients with osteoarthritis and varus deformity (542 knees) who underwent primary TKA at Rajasthan Hospital Limited, Jaipur, Rajasthan, India. A single joint replacement surgeon with approximately 15 years of experience performing primary TKA, particularly in patients with varus knee deformities, performed the index procedures between July 2022 and July 2023. Clinical and radiological records were reviewed through July 2025, yielding 24-36 months of follow-up. Of the 300 patients, 192 were women and 108 were men. All procedures used a posterior-stabilized TKA design, and patellar resurfacing was performed in 60 knees.

Sampling technique and eligibility criteria

A total-enumeration sampling approach was used, with consecutive inclusion of all eligible cases during the predefined surgical period. We included all records that met the eligibility criteria; we did not perform random or selective case sampling.

The inclusion criteria were primary TKA for knee osteoarthritis with varus deformity, with or without associated flexion deformity, and availability of the clinical and radiological data required for the planned analysis. Exclusion criteria were TKA performed for tibiofibular stress fractures, revision TKA, lower-limb valgus deformity, and neutral preoperative alignment corresponding to CPAK type 2.

All eligible consecutive primary TKAs for varus osteoarthritis performed during the predefined study interval were included if they fulfilled the stated eligibility criteria. No eligible case was excluded on the basis of postoperative outcome, intraoperative balancing result, or technical success.

Ethical approval

The study was reviewed and approved by the RHL Ethics Committee (approval number: RHL EC/P 23/2022; approval date: June 7, 2022). All procedures were conducted in accordance with institutional ethical standards and the Declaration of Helsinki and its later amendments. Written informed consent had been obtained from all patients.

Data collection and radiological assessment

CPAK classification was determined from the aHKA and JLO measured on standing anteroposterior long-leg radiographs, using the classification method and category thresholds described by MacDessi et al. [12]. Preoperative and postoperative measurements were classified using the same published CPAK criteria. CPAK preservation was defined as assignment to the same CPAK category at both assessments. No study-specific modification of the published CPAK classification scheme was applied.

Clinical evaluation and scoring systems

Clinical outcome data were extracted from medical records using the KSS [13] and WOMAC [14], while pain was assessed using the VAS [15]. The 2011 Knee Society Scoring System© was downloaded from the official website of the Knee Society and used in its entirety without modification, in accordance with the stated conditions of use. The 2011 Knee Society Scoring System© is used with permission from The Knee Society. © by The Knee Society. All rights reserved and reproduced with permission. For this analysis, postoperative ROM, KSS, and WOMAC values correspond to the standardized 12-month postoperative assessment. Pain during walking or stair use was assessed using the VAS preoperatively and at one month postoperatively.

Socioeconomic status assessment

Socioeconomic status was classified using the Revised Kuppuswamy Socioeconomic Status Scale (2021) [16]. The scale considers the education and occupation of the head of the family along with total monthly family income. It categorizes participants into upper (26-29), upper-middle (16-25), middle (11-15), lower-middle (5-10), and lower (<5) socioeconomic classes based on the composite socioeconomic score. Socioeconomic classification was recorded at the patient level (n = 300).

Surgical technique

All procedures were performed through a midline skin incision and medial parapatellar arthrotomy. A bone-cut-first DMA algorithm was used to obtain functional balance primarily through patient-specific adjustment of bony resections, followed by limited soft-tissue release only when required.

Preoperative Assessment

Preoperative assessment focused on coronal-plane laxity with the knee in full extension and at 90° of flexion. The planned tibial resection was guided by the degree of medial tightness and overall ligament balance. A 7-8 mm resection was planned in knees with minimal or no laxity, whereas a 5-6 mm resection was planned in knees with gross laxity. The CPAK type, angle between the femoral mechanical and anatomical axes, native posterior tibial slope, medial proximal tibial angle (MPTA), and lateral distal femoral angle (LDFA) were recorded from standing anteroposterior scannograms.

Preoperative coronal laxity was assessed manually with varus-valgus stress in full extension and at 90° of flexion. Because instrumented stress measurements were not used, laxity categories were defined clinically. Minimal or no laxity referred to a stable compartment with little appreciable opening during manual stress, whereas gross laxity referred to clearly increased coronal opening during manual assessment. Intraoperatively, marked medial tightness meant preferential resistance of the medial compartment during spacer-block assessment despite acceptable lateral seating. Severe medial tightness was defined as persistent medial resistance with substantial lateral laxity after the initial bone cuts. These categories were based on the operating surgeon’s manual assessment rather than predefined millimetric thresholds.

Surgical Exposure and Joint Preparation

After exposure through the medial parapatellar approach, the menisci and cruciate ligaments were removed as required for a posterior-stabilized construct. The deep medial collateral ligament was elevated from the joint line to permit anterior translation of the proximal tibia, and the posterior cruciate ligament was detached from its femoral insertion and excised.

Tibial Resection

The proximal tibial cut was made using an extramedullary alignment guide oriented to the tibial mechanical axis. In knees with gross preoperative laxity, approximately 5 mm was resected from the base of the lateral tibial spine; in knees with minimal or no laxity, approximately 7 mm was resected. The posterior tibial slope was limited to a maximum of 5° and kept within 3° of the native slope.

Distal Femoral Resection

The distal femoral resection was individualized. The angle between the anatomical and mechanical femoral axes was measured using preoperative standing scannograms and used to set the intramedullary guide. When the extension gap remained selectively tight medially despite an adequate flexion gap, the distal femoral cutting block was adjusted by approximately 1-2° into varus, removing an additional 1-2 mm from the medial distal femoral condyle rather than releasing the superficial medial collateral ligament (sMCL). This targeted varus distal femoral modulation is shown in Figure 1.

**Figure 1:**
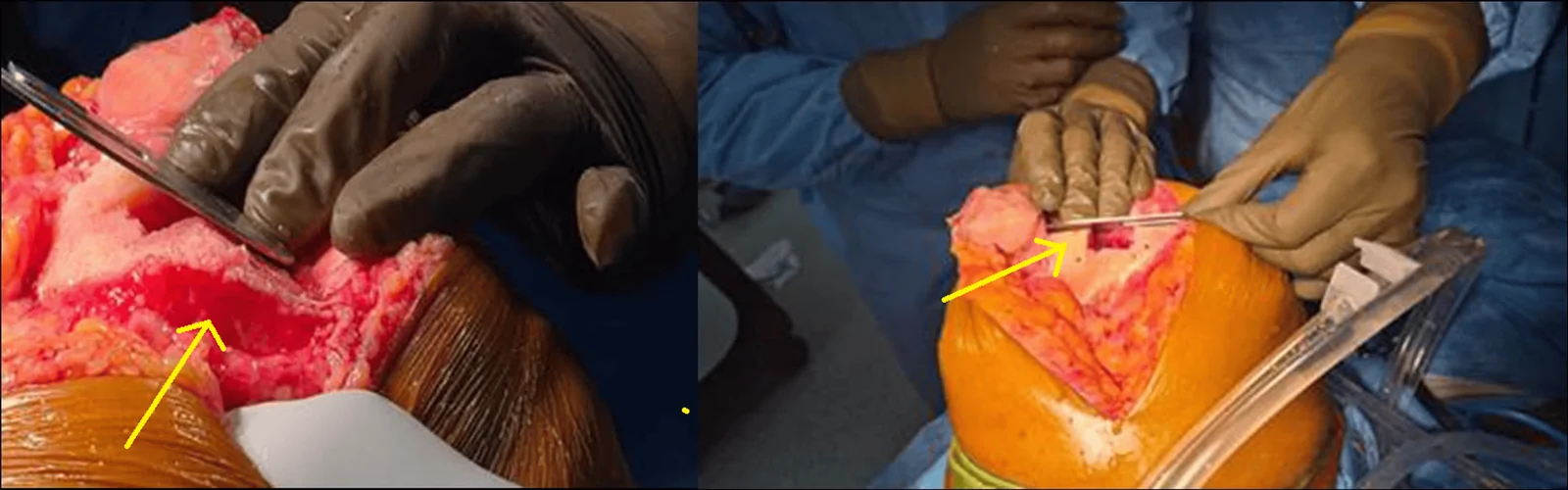
Manual varus distal femoral modulation Yellow arrows indicate the saw blade/cutting orientation used to create the targeted distal femoral varus resection.

Extension Gap Assessment

The extension gap was assessed manually with spacer blocks and interpreted together with the flexion gap. If both compartments were tight, an additional 2 mm tibial resection was performed. If the extension gap was selectively tight medially but the lateral compartment remained lax, correction was deferred until the flexion gap was assessed. When medial tightness was present in both flexion and extension, a controlled varus tibial recut of up to 4° was used to address the medial column across the arc of motion.

Femoral Sizing and Flexion Gap Assessment

After distal femoral resection, the flexion gap was assessed at 90° under consistent manual distraction. One assistant elevated the femur while another drew the tibia distally. Four intraoperative patterns were recognized: a physiologic gap with slight lateral laxity (Figure 2), symmetric tightness (Figure 3), global laxity (Figure 4), and asymmetric medial tightness (Figure 5).

**Figure 2:**
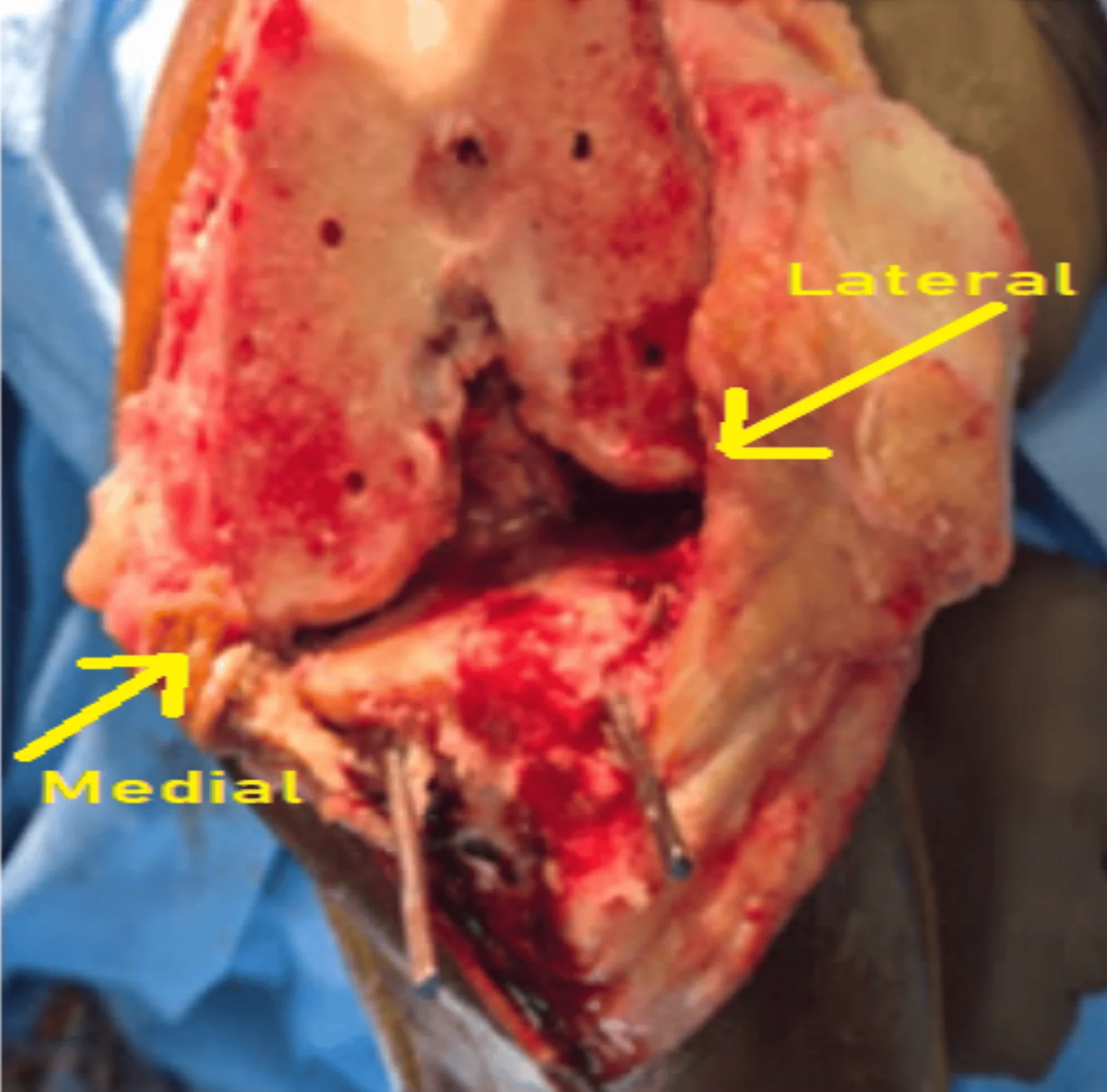
Physiologic flexion-gap pattern Yellow arrows indicate the intraoperative gap pattern with slight lateral compliance relative to the medial side.

**Figure 3:**
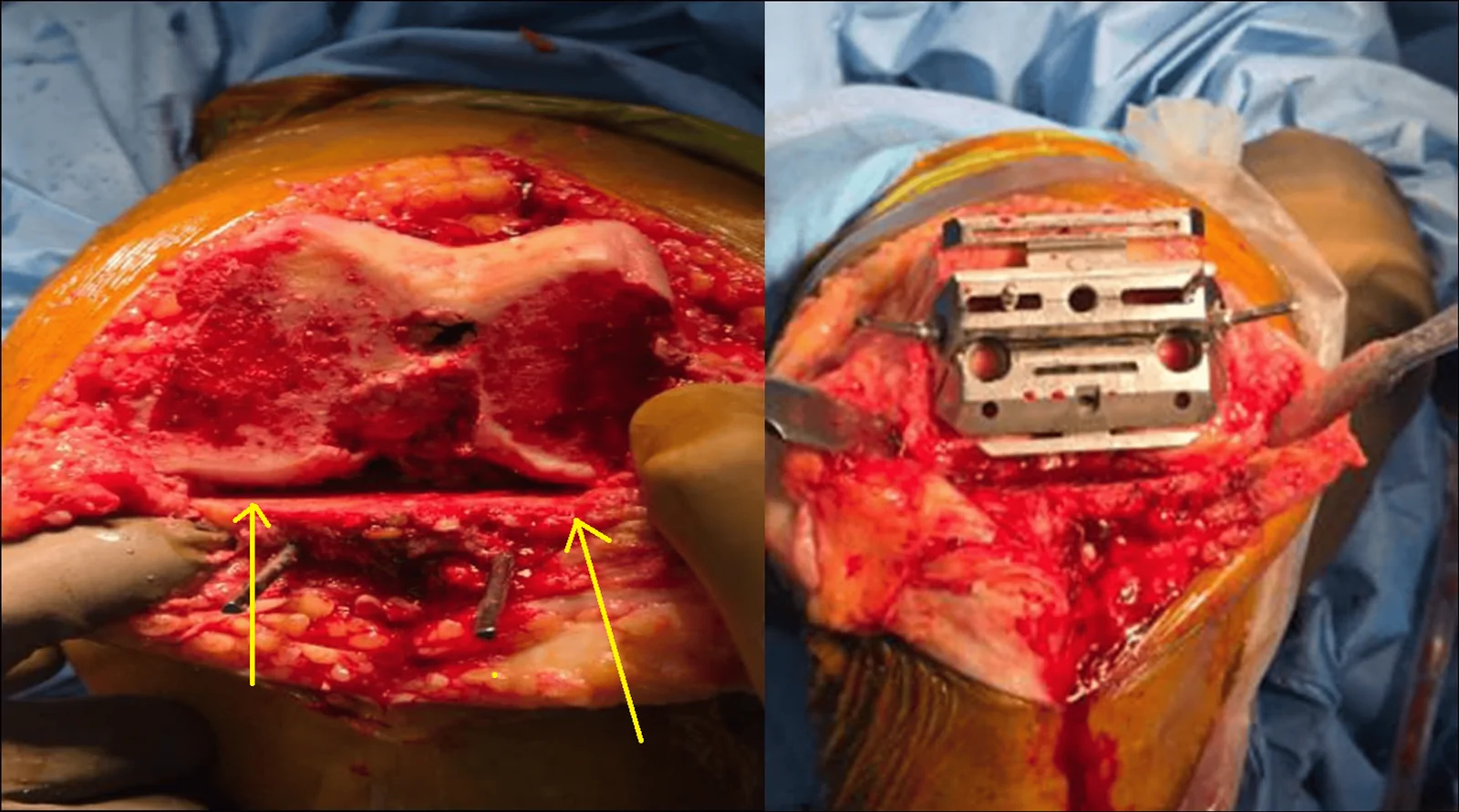
Symmetric tightness Yellow arrows indicate the restricted flexion space in both compartments.

**Figure 4:**
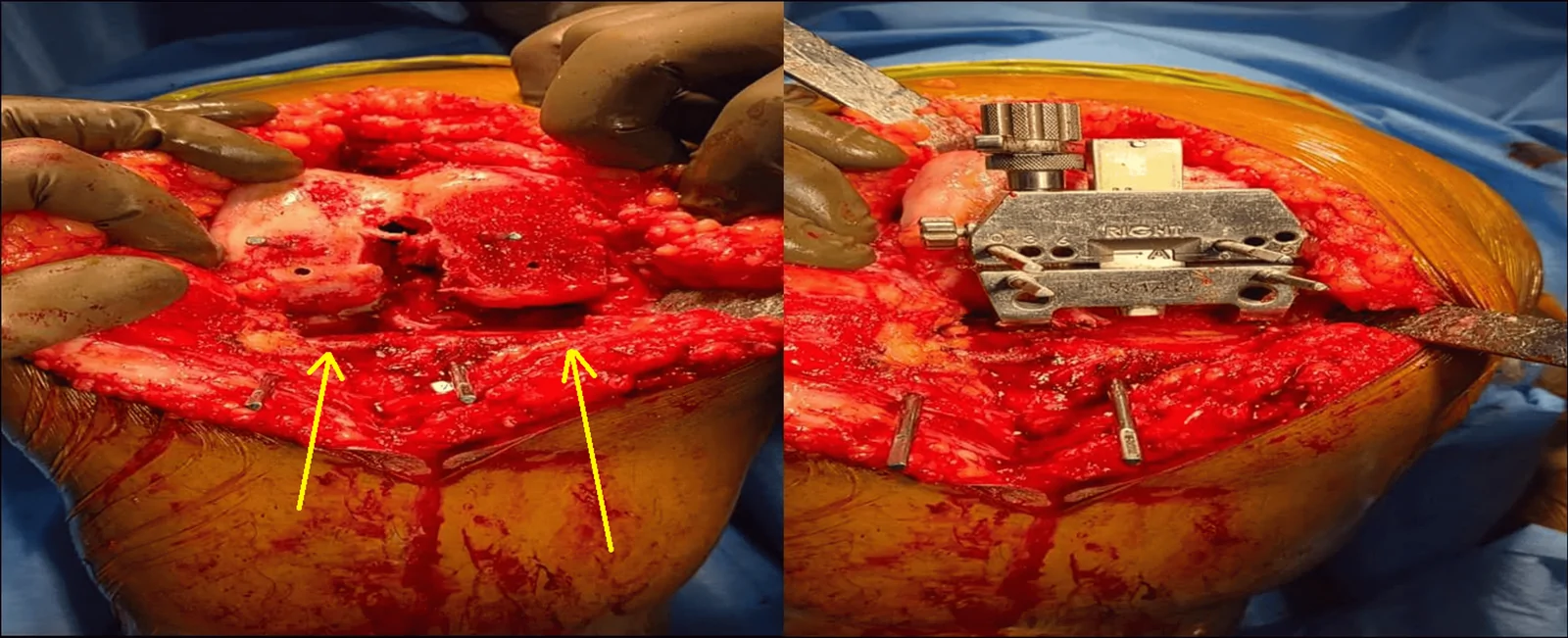
Global laxity Yellow arrows indicate the broadly increased flexion space before final balancing.

**Figure 5:**
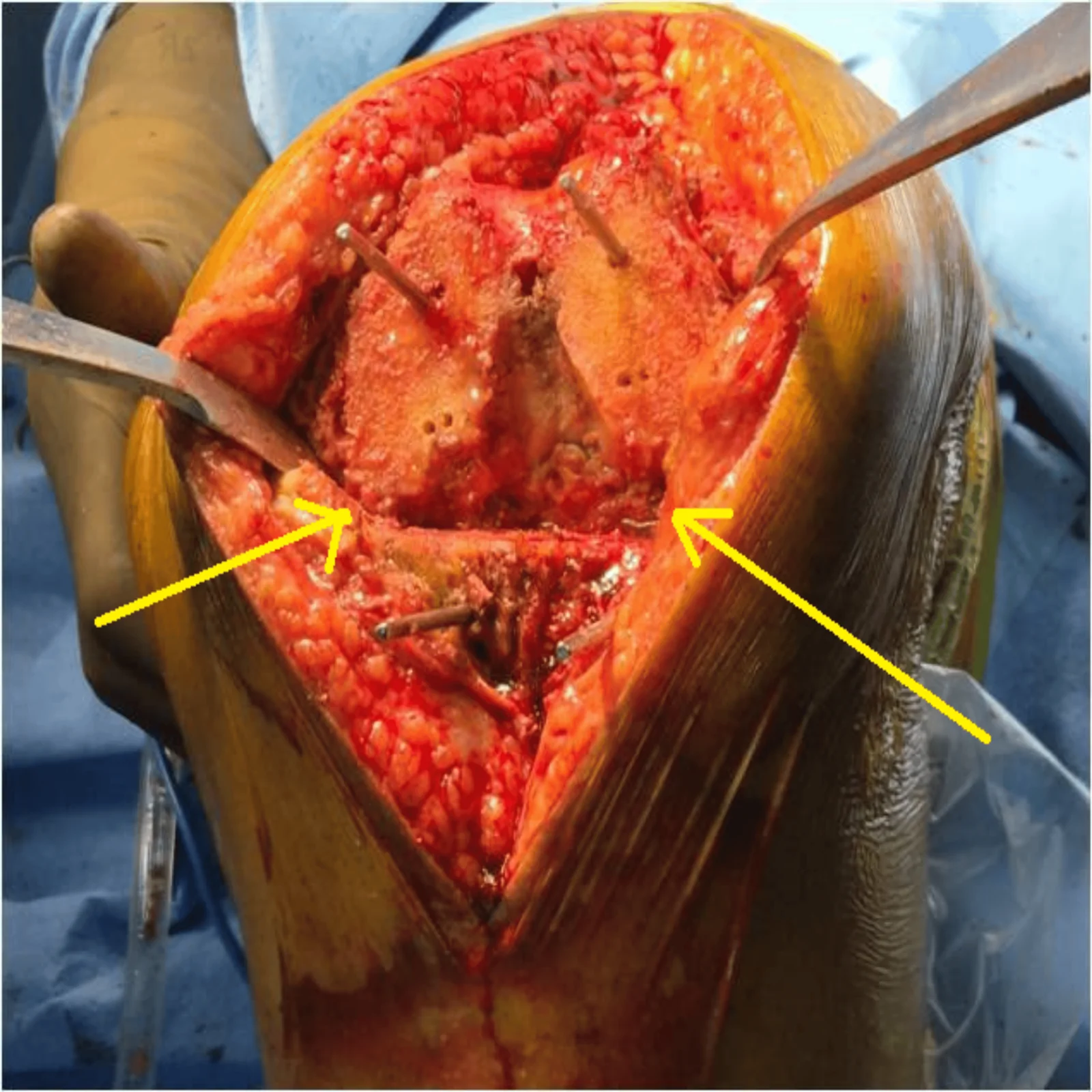
Asymmetric medial tightness Yellow arrows indicate the compartment targeted for restriction during balancing.

Instrument Positioning and Gap Verification

Before posterior femoral resection, a 4-in-1 cutting block was pinned at a baseline 3° of external rotation relative to the posterior condylar axis. A 6 mm spacer was placed between the posterior surface of the jig and the proximal tibial cut to represent the final flexion gap for a 9 mm tibial insert after accounting for the 3 mm offset between the distal surface of the jig and the cutting slot (Figure 6). Equal seating of the spacer medially and laterally, with slight lateral laxity, was considered an acceptable flexion-gap target.

**Figure 6:**
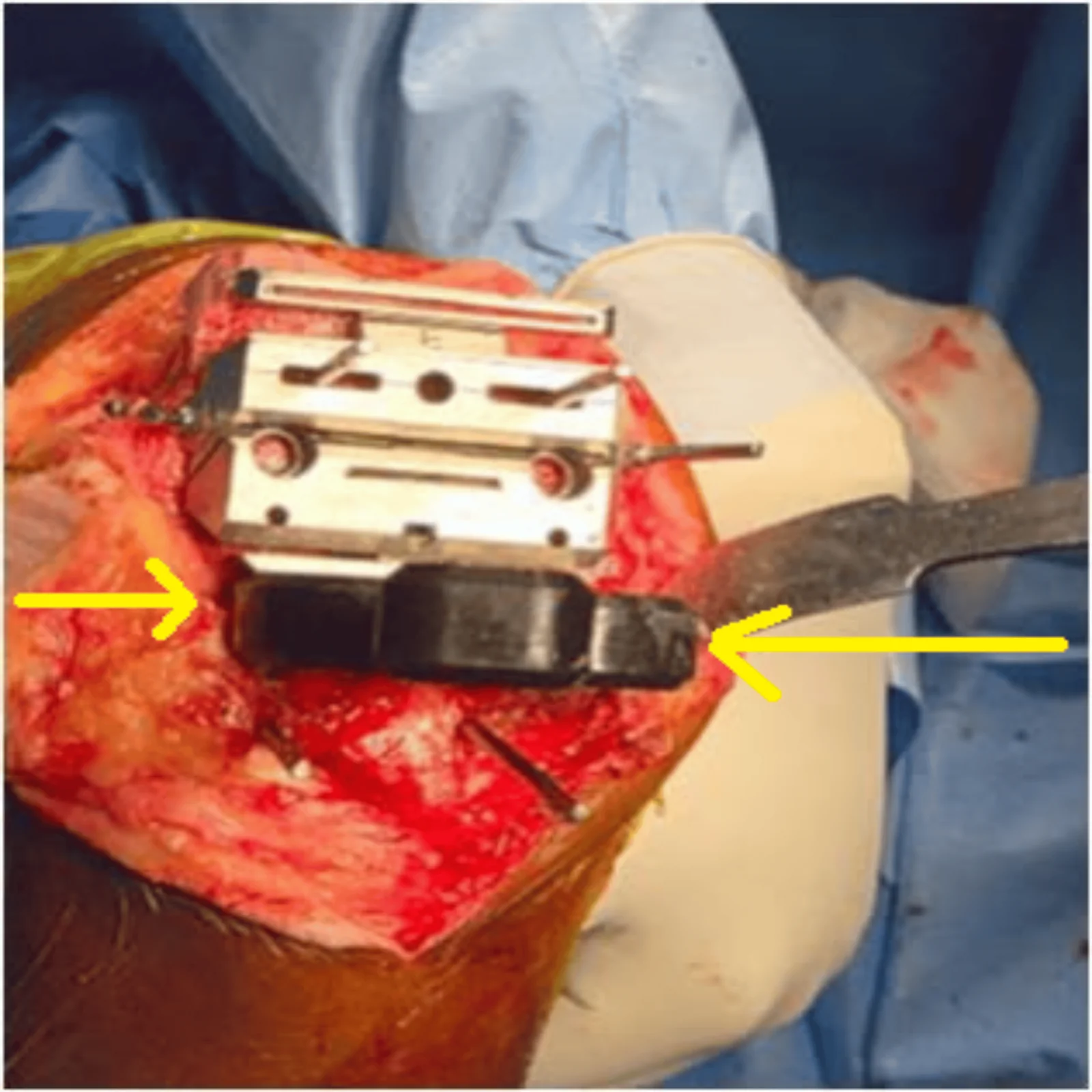
Flexion-gap verification with a 6 mm spacer Yellow arrows indicate the spacer between the posterior surface of the cutting jig and the proximal tibial cut.

Intraoperative Adjustments

If the 6 mm spacer did not seat appropriately, femoral size, anteroposterior position, and component rotation were adjusted rather than proceeding directly to ligament release. When safe, the femoral jig could be translated approximately 2 mm anteriorly, or the component could be downsized. In knees with marked medial flexion tightness, external rotation was increased from 3° to approximately 4.5° (Figure 7). For severe medial flexion tightness with substantial lateral laxity, independent condylar pinning using a 0°/6° strategy was performed rather than applying uniform 3° rotation (Figure 8). In cases of global flexion laxity, the posterior resection was reduced to preserve posterior femoral bone stock.

**Figure 7:**
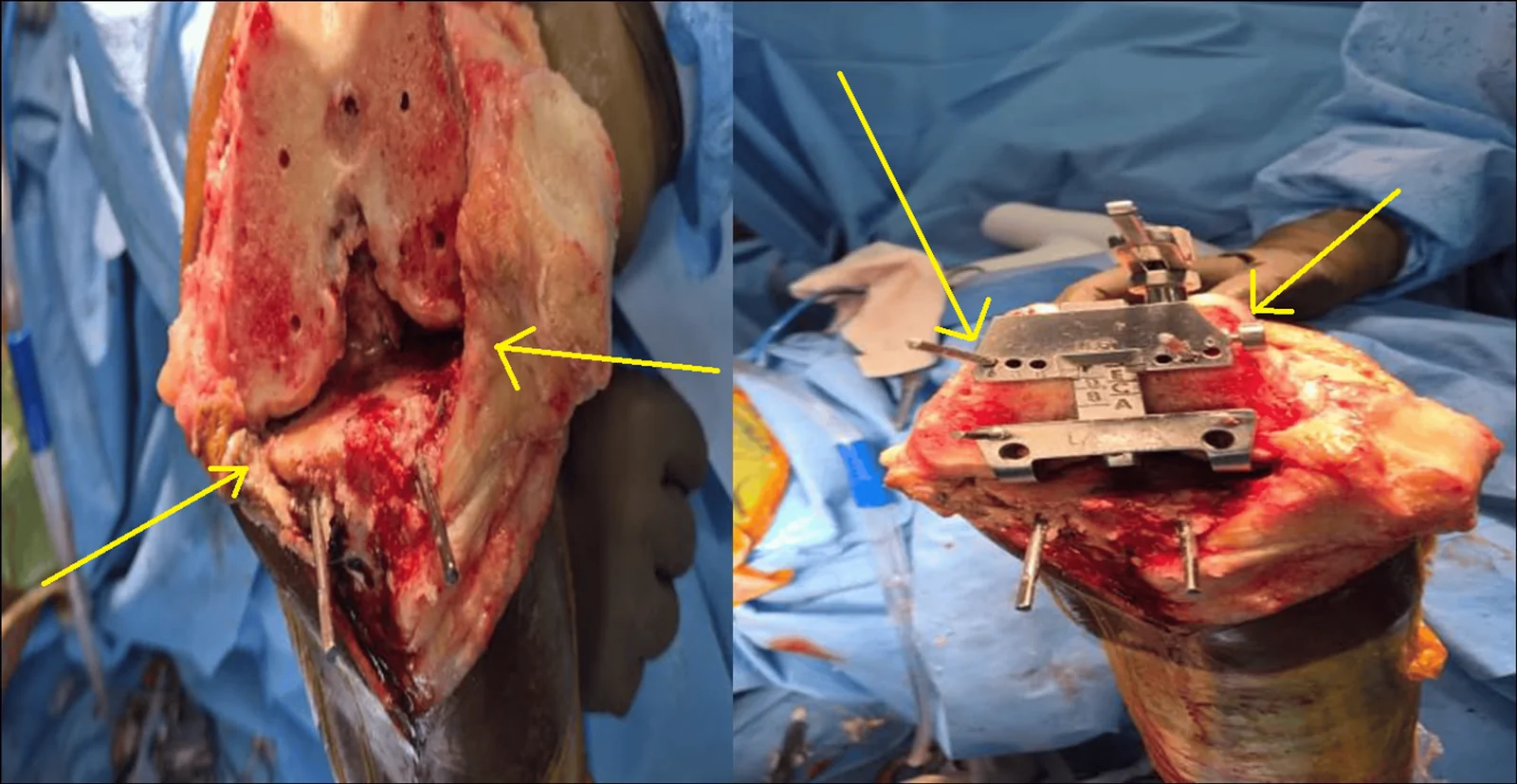
Increased femoral external rotation for marked medial flexion tightness Yellow arrows indicate the adjusted femoral cutting jig set to approximately 4.5° of external rotation.

**Figure 8:**
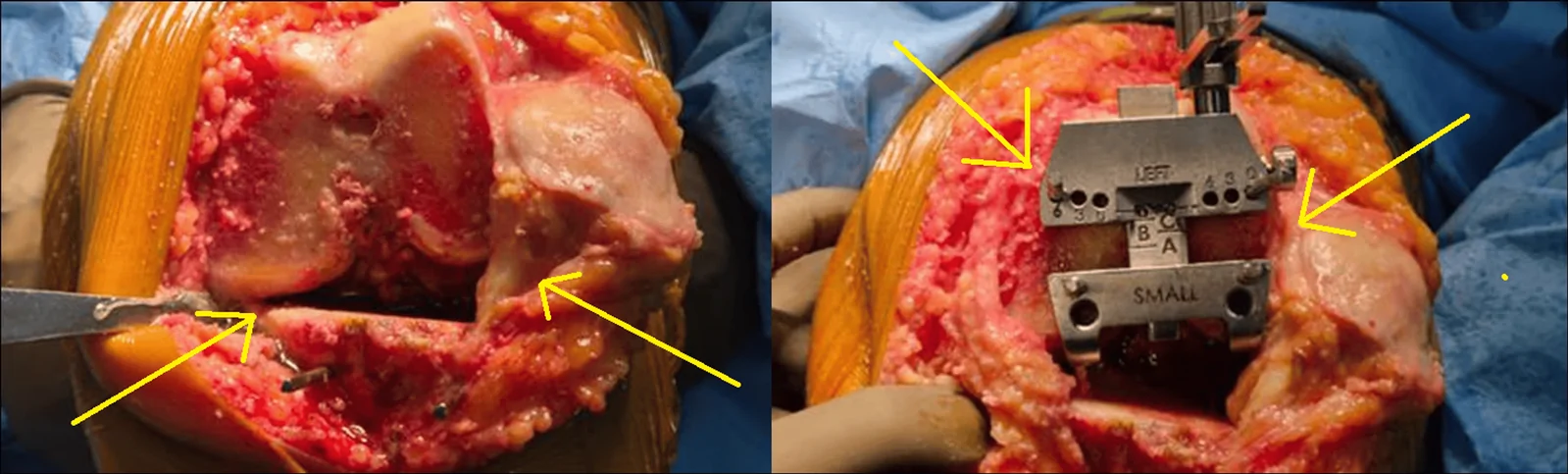
Independent condylar pinning for severe medial flexion tightness with lateral laxity Yellow arrows indicate the 0°/6° rotational-modulation construct.

Tibial Preparation and Final Gap Balancing

The tibia was prepared using an appropriately sized baseplate, with careful attention to rotational alignment. Flexion and extension gaps were then reassessed. When medial osteophytes were present and medial tightness persisted, a medial tibial reduction osteotomy was performed. In the absence of substantial medial osteophytes, a varus tibial recut was performed incrementally using a 1 mm saw blade, with gap reassessment after each cut and a maximum correction of approximately 4° (Figure 9). If medial tightness persisted after bony correction, the semimembranosus tendon was released sequentially posteromedially. The study cohort did not require an sMCL release.

**Figure 9:**
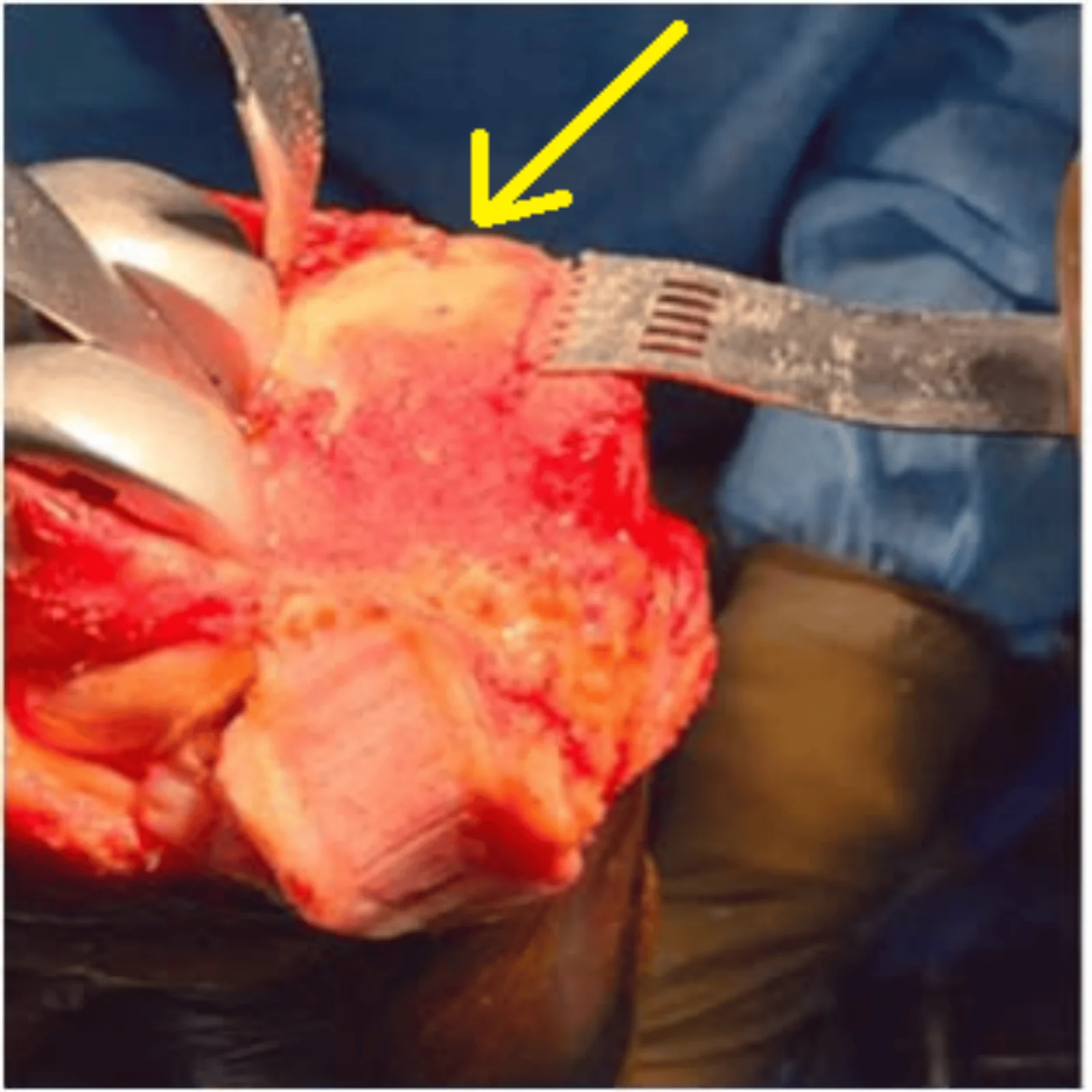
Incremental varus tibial recut The yellow arrow indicates the saw blade position used for controlled coronal correction.

Target Gap Pattern

The final balancing procedure aimed to reproduce physiologic mediolateral asymmetry rather than a completely rectangular or symmetric flexion gap. Balance was assessed manually using spacer blocks and controlled manual distraction. At 90° of flexion, the medial compartment was kept relatively snug while the lateral compartment remained slightly more compliant. At 15-20° of flexion, the target opening was approximately 1 mm medially and 2-3 mm laterally (Figure 10). In full extension, both compartments were expected to remain stable without appreciable opening. Thus, the intended operative endpoint represented controlled physiologic asymmetry rather than equal medial and lateral gap opening. The intended HKA safety boundary was approximately 6° of varus to 3° of valgus, with tibial varus recuts limited to 4°.

**Figure 10:**
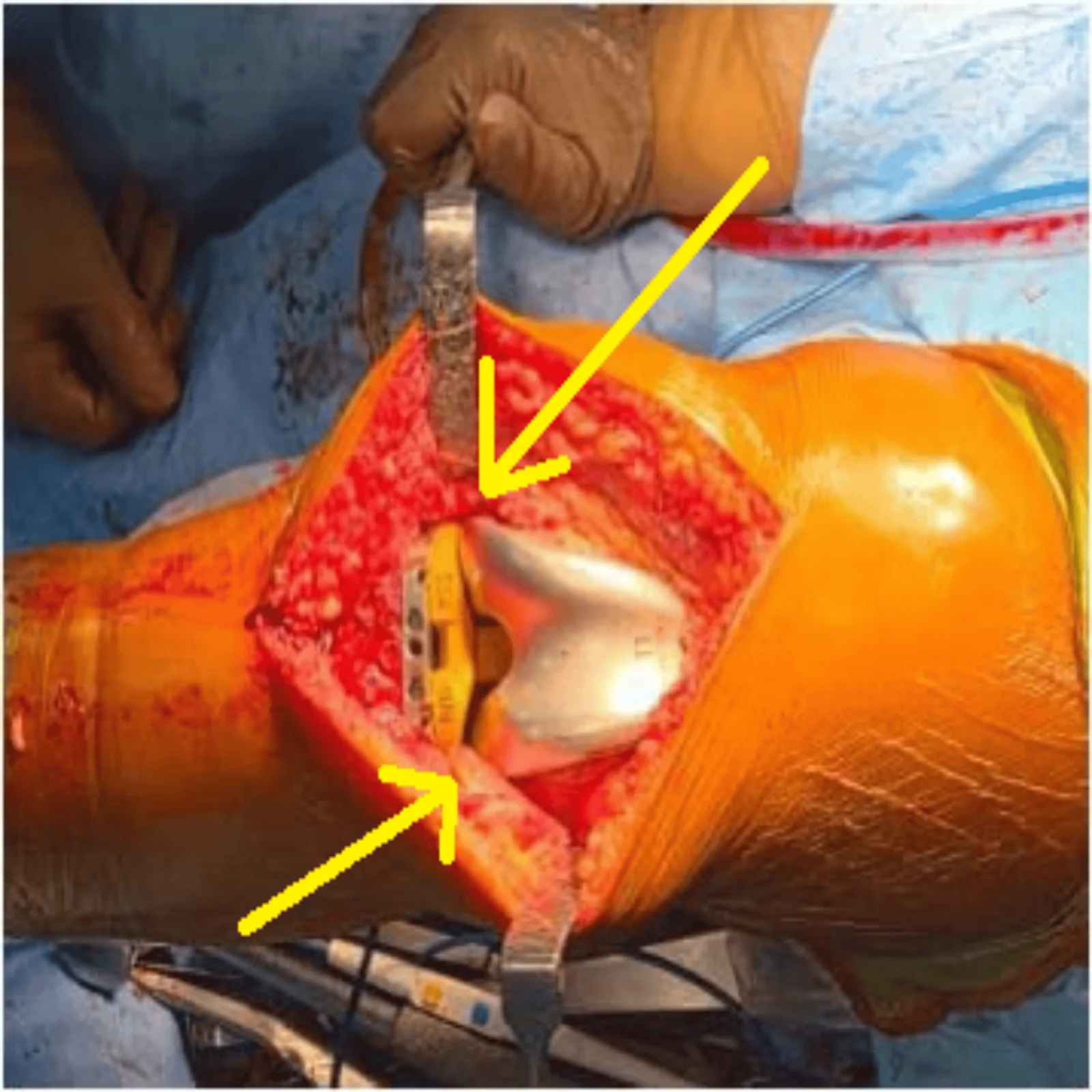
Target gap pattern at 15-20° of flexion Yellow arrows indicate the compartmental opening assessed during final balancing; the intended opening was approximately 1 mm medially and 2-3 mm laterally.

DMA Decision Algorithm

The DMA workflow followed a sequential bone-modulation strategy. Initial proximal tibial resection was selected according to preoperative coronal laxity, with approximately 5 mm resected in knees demonstrating gross laxity and approximately 7 mm in knees with minimal or no laxity. After the initial tibial and distal femoral resections, the extension and flexion gaps were reassessed. If both compartments were tight in extension, an additional 2 mm tibial resection was performed. Persistent medial tightness in both flexion and extension was managed by incremental varus tibial recutting, limited to approximately 4°. When medial tightness was predominantly present in flexion, femoral external rotation could be increased from 3° to approximately 4.5°. Severe medial flexion tightness associated with substantial lateral laxity was managed using independent condylar pinning with a 0°/6° rotational strategy. Global flexion laxity was addressed by reducing posterior femoral resection. After bony correction, persistent medial tightness was managed with medial tibial reduction osteotomy when appropriate, followed by selective semimembranosus release if required. No sMCL release was required in this cohort.

Potential confounders and bias

Potential confounders considered during interpretation included age, sex, baseline coronal alignment and CPAK phenotype, baseline clinical scores, patellar resurfacing, and postoperative complications. No multivariable adjusted model was performed; therefore, residual confounding and the non-independence of bilateral knees were considered when interpreting the findings.

Sample size

Because the study used total enumeration with consecutive inclusion of all eligible cases during the predefined study period, no prospective a priori sample-size calculation was performed. The final analyzable dataset comprised all 300 eligible patients, representing 542 operated knees.

Statistical analysis

Continuous variables were summarized as mean ± standard deviation (SD), and categorical variables were summarized as counts and percentages. Preoperative and postoperative continuous outcomes were compared using paired-samples t-tests. Continuous variables were compared between the CPAK-changed and CPAK-unchanged groups using independent-samples t-tests. Categorical variables were analyzed using Pearson's chi-square test, with Fisher's exact test used when expected cell counts were small. All tests were two-sided, and p < 0.05 was considered statistically significant. Data management and statistical calculations were performed using Excel (Microsoft Corporation, Redmond, Washington, USA).

Because some participants underwent bilateral TKA, knee-level observations were not completely independent. The retrospective dataset was analyzed using paired-samples and independent-samples t-tests, and no mixed-effects, generalized estimating equation, or other cluster-adjusted analysis was performed. Accordingly, the reported p-values should be interpreted cautiously, particularly for knee-level between-group comparisons. Given the exploratory nature of the secondary analyses and the number of comparisons performed, statistical significance was interpreted together with the magnitude and clinical relevance of the observed differences.

## Results

Participant characteristics

The study included 300 patients with 542 operated knees. The mean age was 67.36 ± 6.64 years (range: 44-82 years). The sample included 192 women (64.0%) and 108 men (36.0%). According to the Revised Kuppuswamy Socioeconomic Status Scale (2021), 87 participants (29.0%) belonged to the upper socioeconomic class, 209 (69.7%) to the upper-middle class, and four (1.3%) to the middle class. No participant belonged to the lower-middle or lower socioeconomic classes. Thus, 296 participants (98.7%) belonged to the upper or upper-middle socioeconomic strata. Patellar resurfacing was performed in 60 knees. Clinical and radiological follow-up ranged from 24 to 36 months. Participant characteristics are summarized in Table 1.

**Table 1:** Participant demographic and socioeconomic characteristics Age, sex, and socioeconomic status are reported at the patient level (n = 300), whereas patellar resurfacing is reported at the knee level (n = 542). Socioeconomic status was classified using the Revised Kuppuswamy Socioeconomic Status Scale (2021). SD: standard deviation

Characteristic	Category	Value
Number of patients	-	300
Number of operated knees	-	542
Age, years	Mean ± SD	67.36 ± 6.64
Age, years	Range	44-82
Sex	Male	108 (36.0%)
Sex	Female	192 (64.0%)
Socioeconomic class	Upper, score 26-29	87 (29.0%)
Socioeconomic class	Upper-middle, score 16-25	209 (69.7%)
Socioeconomic class	Middle, score 11-15	4 (1.3%)
Socioeconomic class	Lower-middle, score 5-10	0 (0.0%)
Socioeconomic class	Lower, score <5	0 (0.0%)
Patellar resurfacing	Performed	60 knees
Follow-up	Range	24-36 months

Clinical and radiological outcomes

The principal clinical outcome measures improved significantly at the standardized 12-month postoperative assessment. Mean ROM increased from 95.66 ± 11.56° preoperatively to 133.82 ± 7.69° at 12 months (paired-samples t-test: t = 18.42, p < 0.001). Mean KSS increased from 24.70 ± 14.63 to 85.58 ± 8.35 (t = 25.76, p < 0.001), while mean WOMAC decreased from 62.62 ± 7.01 to 10.82 ± 13.00 (t = 24.31, p < 0.001).

Mean aHKA changed from −8.26 ± 1.78° preoperatively to −1.58 ± 0.98° at 12 months (t = 22.64, p < 0.001). The mean LDFA changed from 88.20 ± 0.92° to 89.24 ± 0.91°, and the mean MPTA changed from 81.52 ± 1.73° to 86.12 ± 1.43°. Table 2 summarizes the principal preoperative and 12-month postoperative outcomes.

**Table 2:** Preoperative and 12-month postoperative clinical and radiological outcomes Postoperative values correspond to the standardized 12-month postoperative assessment. Preoperative and postoperative values were compared using paired-samples t-tests. Because some participants underwent bilateral TKA, knee-level observations were not completely independent, and no cluster-adjusted analysis was performed; inferential results should therefore be interpreted cautiously. Data are expressed as mean ± SD. Preoperative and postoperative values were compared using paired-samples t-tests. The corresponding t-statistic and two-sided p-value are reported for each comparison. A p-value <0.05 was considered statistically significant. ROM: range of motion, KSS: Knee Society Score, WOMAC: Western Ontario and McMaster Universities Osteoarthritis Index, aHKA: arithmetic hip-knee-ankle angle, LDFA: lateral distal femoral angle, MPTA: medial proximal tibial angle

Outcome	Preoperative	Postoperative	Test statistic	p-value
ROM (°)	95.66 ± 11.56	133.82 ± 7.69	t = 18.42	<0.001
KSS	24.70 ± 14.63	85.58 ± 8.35	t = 25.76	<0.001
WOMAC	62.62 ± 7.01	10.82 ± 13.00	t = 24.31	<0.001
aHKA (°)	−8.26 ± 1.78	−1.58 ± 0.98	t = 22.64	<0.001
LDFA (°)	88.20 ± 0.92	89.24 ± 0.91	t = 18.65	<0.001
MPTA (°)	81.52 ± 1.73	86.12 ± 1.43	t = 42.31	<0.001

The mean VAS pain score during walking or stair use decreased from 6.47 ± 0.99 preoperatively to 1.82 ± 0.87 at one month postoperatively.

CPAK classification and between-group comparison

Of the 542 operated knees, 418 (77.12%) retained their preoperative CPAK class, whereas 124 knees (22.88%) changed CPAK class postoperatively. CPAK preservation was evaluated as a descriptive radiological outcome and was not interpreted as evidence of superior clinical outcome. Postoperative ROM was higher in the CPAK-changed group than in the CPAK-unchanged group. In contrast, postoperative KSS, WOMAC, VAS, aHKA, JLO, MPTA, and LDFA did not differ significantly between groups in the original analysis. Table 3 presents detailed comparisons.

**Table 3:** Clinical and radiological parameters according to postoperative change in CPAK classification Data are expressed as mean ± SD. Between-group comparisons were performed using independent-samples t-tests. Postoperative ROM, KSS, WOMAC, aHKA, JLO, MPTA, and LDFA values correspond to the 12-month postoperative assessment, while postoperative VAS corresponds to the one-month assessment. Because some patients contributed both knees, complete independence of knee-level observations could not be assumed. No cluster-adjusted analysis was performed; therefore, these comparisons should be regarded as exploratory and interpreted cautiously. CPAK preservation was treated as a descriptive radiological outcome and not as a validated surrogate for superior clinical outcome. A p-value <0.05 was considered statistically significant. CPAK: coronal plane alignment of the knee, ROM: range of motion, KSS: Knee Society Score, WOMAC: Western Ontario and McMaster Universities Osteoarthritis Index, VAS: Visual Analog Scale, aHKA: arithmetic hip-knee-ankle angle, JLO: joint-line obliquity, MPTA: medial proximal tibial angle, LDFA: lateral distal femoral angle

Parameter	CPAK-changed (n = 124)	CPAK-unchanged (n = 418)	Test statistic	p-value
ROM, preoperative (°)	96.67 ± 4.75	96.03 ± 12.45	t = 0.41	0.683
ROM, 12 months (°)	136.67 ± 4.75	133.28 ± 8.14	t = 3.31	0.001
KSS, preoperative	27.47 ± 8.56	21.94 ± 14.50	t = 0.81	0.421
KSS, 12 months	86.67 ± 4.79	84.67 ± 8.70	t = 1.82	0.069
WOMAC, preoperative	65.50 ± 4.02	62.50 ± 7.39	t = 1.70	0.089
WOMAC, 12 months	10.67 ± 2.38	10.83 ± 14.47	t = −0.09	0.926
VAS, preoperative	7.67 ± 0.48	8.59 ± 1.02	t = −0.07	0.945
VAS, 1 month	1.67 ± 0.95	1.92 ± 0.85	t = −1.84	0.066
aHKA, preoperative (°)	−9.17 ± 1.47	−8.33 ± 1.73	t = −1.70	0.089
aHKA, 12 months (°)	−1.33 ± 0.67	−1.92 ± 0.76	t = 1.68	0.093
JLO, preoperative (°)	170.17 ± 2.75	169.58 ± 1.86	t = 1.78	0.076
JLO, 12 months (°)	174.83 ± 2.28	174.22 ± 1.87	t = 1.71	0.087
MPTA, preoperative (°)	81.67 ± 1.90	81.38 ± 1.71	t = 1.25	0.212
MPTA, 12 months (°)	86.33 ± 1.26	86.05 ± 1.51	t = 1.46	0.144
LDFA, preoperative (°)	88.50 ± 1.27	89.12 ± 0.88	t = −1.72	0.086
LDFA, 12 months (°)	88.50 ± 1.27	89.17 ± 0.87	t = −1.76	0.078

Postoperative events

Forty patients (67 operated knees; 12.36%) experienced transient postoperative stiffness or gelling symptoms, particularly after waking or prolonged sitting. These symptoms resolved within three months in all affected patients. One patient required manipulation under anesthesia of both knees at six weeks because ROM was limited to 0-60°. Twenty-one patients required a handrail for support while climbing or descending stairs, and one patient had a residual 10° flexion deformity.

Twelve patients developed superficial wound complications, including superficial infection or stitch abscess. One patient developed a deep infection requiring surgical debridement and antibiotic therapy, and one patient experienced pulmonary embolism. No patient developed symptoms attributable to patellar maltracking.

Postoperative complications and adverse events, with patient-level denominators and knee-level involvement where applicable, are summarized in Table 4.

**Table 4:** Postoperative complications and adverse events Complication frequencies are reported at the patient level using 300 patients as the denominator and, where applicable, at the knee level using 542 operated knees as the denominator. Laterality is reported where available. Pulmonary embolism was considered a systemic patient-level event and therefore does not have a knee-level denominator.

Event	Patients, n (%)	Knee-level information
Transient postoperative stiffness or gelling symptoms	40/300 (13.3%)	67/542 knees (12.36%)
Manipulation under anesthesia	1/300 (0.3%)	2/542 knees (0.37%); bilateral
Superficial wound complication, including superficial infection or stitch abscess	12/300 (4.0%)	12/542 knees (2.21%); left: 8, right: 4
Deep infection requiring debridement and antibiotic therapy	1/300 (0.3%)	1/542 knees (0.18%); left knee
Pulmonary embolism	1/300 (0.3%)	Not applicable; systemic patient-level event
Residual 10° flexion deformity	1/300 (0.3%)	1/542 knees (0.18%); left knee
Symptomatic patellar maltracking	0/300 (0%)	0/542 knees (0%)

## Discussion

This retrospective study evaluated the clinical and radiological outcomes of a manual DMA approach in 300 patients with 542 varus knees undergoing primary TKA. At the standardized 12-month assessment, ROM, KSS, WOMAC, and radiological parameters improved, and early pain scores improved at one month. The intended physiologic mediolateral balance pattern was achieved without sMCL release, and 418 of 542 knees (77.12%) retained their preoperative CPAK class. These findings demonstrate the technical feasibility of the described manual bone-modulation approach but do not establish comparative clinical effectiveness.

CMA has historically been the most widely used alignment strategy in TKA because of its reproducibility and association with long-term implant survivorship. However, some patients remain dissatisfied despite technically successful mechanically aligned TKA, so attention has increasingly shifted toward individualized alignment strategies [17]. CMA applies relatively uniform coronal alignment targets across patients, whereas contemporary personalized approaches attempt to account for differences in native anatomy, joint-line orientation, and soft-tissue balance.

DMA was developed as a manual bone-modulation strategy intended to individualize alignment and gap balancing while maintaining defined mechanical limits. Rather than relying exclusively on fixed resection angles followed by extensive soft-tissue releases, the technique allows controlled adjustment of distal femoral resection, femoral component rotation, proximal tibial coronal alignment, and posterior tibial slope based on the intraoperative soft-tissue envelope. In the present series, this approach allowed balancing without sMCL release.

Relationship to functional alignment

FA remains variably defined in the arthroplasty literature, and several authors have emphasized the need for more consistent terminology and reporting [18]. Computer-assisted and robotic platforms have been used to describe techniques incorporating individualized component positioning and intraoperative soft-tissue balancing [19,20]. DMA shares several principles with FA, particularly individualized bony resections, adjustment according to ligamentous balance, and avoidance of unnecessary soft-tissue releases. The principal distinction is that DMA is performed manually and within predefined coronal alignment boundaries.

In the present technique, the overall alignment strategy remained restricted rather than unrestricted. Femoral rotation was maintained within a posterior condylar axis-based external-rotation range, while tibial coronal correction and posterior slope were adjusted incrementally according to intraoperative balance. Bellemans et al. showed that constitutional alignment varies considerably among individuals and that neutral mechanical alignment does not necessarily reflect each knee's native alignment [21]. This observation supports individualized alignment strategies that preserve patient-specific anatomy within safe operative boundaries.

In the current cohort, 418 knees (77.12%) retained their preoperative CPAK class. CPAK preservation was evaluated as a descriptive radiological finding rather than as a surrogate marker of superior clinical outcome. Although the study explored differences between CPAK-changed and CPAK-unchanged knees, it was not designed to determine whether preservation or alteration of the CPAK class leads to better clinical outcomes. Therefore, these findings should be interpreted cautiously.

Physiologic gap balance

Traditional TKA balancing has often aimed to create symmetric medial and lateral gaps in flexion and extension. However, the native knee exhibits compartment-specific laxity, with greater lateral mobility during flexion than medial mobility [22]. Consequently, complete mediolateral symmetry throughout the ROM may not necessarily reproduce normal knee behavior.

The DMA technique used in this study therefore targeted relative medial stability and controlled lateral compliance during flexion, rather than an absolutely rectangular flexion gap. Previous studies have linked postoperative knee stability, soft-tissue balance, and patient satisfaction [23], while Meneghini et al. have highlighted the potential importance of preserving physiologic asymmetry in primary TKA [24,25]. These findings are consistent with the concept that individualized gap balancing can be achieved without routine, extensive ligamentous release.

Bone modulation and posterior tibial slope

Posterior tibial slope represents another important variable affecting flexion-gap mechanics. Howard et al. emphasized the importance of reproducing the native posterior tibial slope within a limited range during TKA [26]. In the present DMA protocol, postoperative slope was maintained within approximately 3° of the patient's native slope, with an upper limit established to prevent excessive posterior inclination.

Persistent medial tightness was managed sequentially through targeted bony correction. Medial tibial reduction osteotomy or controlled varus tibial recutting was performed before consideration of selective soft-tissue release. When required, semimembranosus release was performed stepwise. This sequence is consistent with the principle of graded posteromedial balancing described by Mullaji et al. [27]. No sMCL release was required in the current cohort.

Clinical outcomes and postoperative stiffness

At the standardized 12-month postoperative assessment, substantial improvements were observed in the principal clinical outcome measures. Mean ROM increased from 95.66° preoperatively to 133.82° at 12 months, while KSS and WOMAC also improved. The mean VAS pain score decreased from 6.47 preoperatively to 1.82 at one month postoperatively. Because the study lacked a comparator group, these postoperative changes should be interpreted as associations observed after TKA performed using the DMA technique rather than effects specifically attributable to DMA.

Targeted bone modulation may reduce the need for extensive soft-tissue release in selected varus knees. Excessive constraint or imbalance after TKA has been associated with postoperative discomfort and restricted motion [28]. In the present cohort, 40 patients with 67 knees experienced transient postoperative stiffness or gelling symptoms that resolved within three months. One patient required manipulation under anesthesia of both knees at six weeks, and one patient had a residual 10° flexion deformity. Published studies have reported postoperative stiffness after TKA at variable rates [29]. However, because the present study lacked a control group, the observed frequency of stiffness cannot be attributed specifically to the DMA technique.

Joint-line obliquity and coronal phenotype

JLO is an additional biomechanical consideration in individualized alignment strategies. Prior biomechanical work has suggested that kinematically aligned TKA may reduce the knee adduction moment compared with mechanically aligned TKA [30]. In the present cohort, the postoperative JLO and CPAK distributions indicated that the manual DMA strategy frequently maintained or approximated the patient's preoperative coronal phenotype. Nevertheless, gait kinetics were not measured directly, and no conclusions regarding gait biomechanics can therefore be drawn from the current data.

Conceptual comparison of CMA and DMA

Table 5 summarizes the conceptual differences between CMA and DMA. The following comparison describes differences in operative philosophy and workflow only. The present study did not directly compare CMA and DMA and therefore does not establish comparative clinical effectiveness.

**Table 5:** Conceptual comparison of CMA and DMA This table summarizes the operative principles described in this manuscript and should not be interpreted as a comparative clinical-effectiveness analysis. CMA: conventional mechanical alignment, DMA: dynamic mechanical alignment, HKA: hip-knee-ankle angle

Feature	CMA	DMA
Alignment philosophy	Standardized mechanical targets	Individualized alignment within restricted mechanical boundaries
Soft-tissue management	Ligament release may be required to achieve balance	Bone modulation prioritized before selective soft-tissue release
Bony resection strategy	Predetermined standardized resections	Patient-specific incremental adjustments
HKA boundary	Target near neutral mechanical alignment	Restricted individualized range
Intraoperative balancing	Balance assessed after standardized cuts	Gap assessment guides subsequent bone modulation
Instrumentation	Conventional manual instrumentation	Conventional manual instrumentation

Several limitations should be considered. The retrospective, single-center design is susceptible to selection and information bias. Although all eligible consecutive cases meeting the predefined criteria were included, this does not eliminate selection bias. All procedures were performed by a single joint replacement surgeon with approximately 15 years of TKA experience, which may introduce operator-dependent effects and limit generalizability to surgeons with different levels of experience. The study included only patients with varus knee morphotypes; therefore, the findings should not be extrapolated to neutral or valgus knees. Intraoperative ligamentous laxity and gap balance were assessed manually rather than using instrumented sensors or predefined quantitative thresholds, which may introduce operator-dependent variability and limit reproducibility. The study did not include a concurrent CMA, KA, or robotic FA comparator; therefore, postoperative improvements cannot be attributed specifically to DMA or interpreted as evidence of superiority. Some participants underwent bilateral TKA, and knee-level analyses did not account for within-patient correlation; therefore, the precision of the reported inferential statistics may be overestimated. Potential confounding variables were not fully controlled, and residual confounding remains possible. Gait analysis and patient-specific biomechanical testing were not performed. Multiple clinical and radiological comparisons were undertaken, and statistical significance should therefore be interpreted together with the magnitude and clinical relevance of the observed differences. Finally, follow-up of 24-36 months is insufficient to assess long-term implant survivorship, aseptic loosening, polyethylene wear, or durability of the observed outcomes.

## Conclusions

In this retrospective single-center cohort of 300 patients with 542 varus knees, TKA performed using the manual DMA technique was associated with improved ROM, KSS, and WOMAC at the standardized 12-month postoperative assessment, along with improved early pain scores at one month. The intended physiologic mediolateral balance pattern was achieved without sMCL release, and 418 knees (77.12%) retained their preoperative CPAK class. These findings demonstrate the technical feasibility of the described manual bone-modulation approach but do not establish superiority over CMA or other individualized alignment strategies. Prospective comparative studies with longer follow-up and statistical methods that account for patient-level clustering are needed to determine comparative clinical effectiveness, reproducibility, and long-term implant outcomes.
